# CCSD(T) Rotational Constants for Highly Challenging C_5_H_2_ Isomers—A Comparison between Theory and Experiment

**DOI:** 10.3390/molecules28186537

**Published:** 2023-09-09

**Authors:** Venkatesan S. Thimmakondu, Amir Karton

**Affiliations:** 1 Department of Chemistry and Biochemistry, San Diego State University, San Diego, CA 92182-1030, USA; 2 School of Science and Technology, University of New England, Armidale, NSW 2351, Australia

**Keywords:** C_5_H_2_, interstellar medium, astrochemistry, rotational constants, CCSD(T), DFT

## Abstract

We evaluate the accuracy of CCSD(T) and density functional theory (DFT) methods for the calculation of equilibrium rotational constants ($A_{e}$, $B_{e}$, and $C_{e}$) for four experimentally detected low-lying C${}_{5}$H${}_{2}$ isomers (ethynylcyclopropenylidene (**2**), pentatetraenylidene (**3**), ethynylpropadienylidene (**5**), and 2-cyclopropen-1-ylidenethenylidene (**8**)). The calculated rotational constants are compared to semi-experimental rotational constants obtained by converting the vibrationally averaged experimental rotational constants ($A_{0}$, $B_{0}$, and $C_{0}$) to equilibrium values by subtracting the vibrational contributions (calculated at the B3LYP/jun-cc-pVTZ level of the theory). The considered isomers are closed-shell carbenes, with cumulene, acetylene, or strained cyclopropene moieties, and are therefore highly challenging from an electronic structure point of view. We consider both frozen-core and all-electron CCSD(T) calculations, as well as a range of DFT methods. We find that calculating the equilibrium rotational constants of these C${}_{5}$H${}_{2}$ isomers is a difficult task, even at the CCSD(T) level. For example, at the all-electron CCSD(T)/cc-pwCVTZ level of the theory, we obtain percentage errors ≤0.4% ($C_{e}$ of isomer **3**, $B_{e}$ and $C_{e}$ of isomer **5**, and $B_{e}$ of isomer **8**) and 0.9–1.5% ($B_{e}$ and $C_{e}$ of isomer **2**, $A_{e}$ of isomer **5**, and $C_{e}$ of isomer **8**), whereas for the $A_{e}$ rotational constant of isomers **2** and **8** and $B_{e}$ rotational constant of isomer **3**, high percentage errors above 3% are obtained. These results highlight the challenges associated with calculating accurate rotational constants for isomers with highly challenging electronic structures, which is further complicated by the need to convert vibrationally averaged experimental rotational constants to equilibrium values. We use our best CCSD(T) rotational constants (namely, ae-CCSD(T)/cc-pwCVTZ for isomers **2** and **5**, and ae-CCSD(T)/cc-pCVQZ for isomers **3** and **8**) to evaluate the performance of DFT methods across the rungs of Jacob’s Ladder. We find that the considered pure functionals (BLYP-D3BJ, PBE-D3BJ, and TPSS-D3BJ) perform significantly better than the global and range-separated hybrid functionals. The double-hybrid DSD-PBEP86-D3BJ method shows the best overall performance, with percentage errors below 0.5% in nearly all cases.

## 1. Introduction

Rotational spectroscopy is one of the most powerful spectroscopic tools for accurately identifying the structural information of molecules in the gas phase [1,2]. Prediction of accurate rotational constants using quantum chemical methods facilitates the laboratory analysis of rotational spectroscopists by carrying out the laboratory search in a relatively narrow range and consequently identifying the detection of unknown molecules [3,4,5,6,7,8,9,10,11,12,13]. However, in order to predict the rotational constants with sufficient accuracy, high levels of the theory, which can be computationally quite demanding, have to be employed [14,15,16]. To be precise, the percent error between the theory and an experiment should not be larger than 0.2% [17,18]. Recent theoretical studies by Puzzarini and Stanton advocate for 0.1% accuracy in order to be useful to rotational spectroscopists, in particular for small- to medium-sized molecules (that is, up to 20 atoms) [19]. These very small percent errors are required, since larger percent errors lead to a wider range in the laboratory search, which is not only cumbersome, but may also lead to inconclusive outcomes.

**Figure 1:**
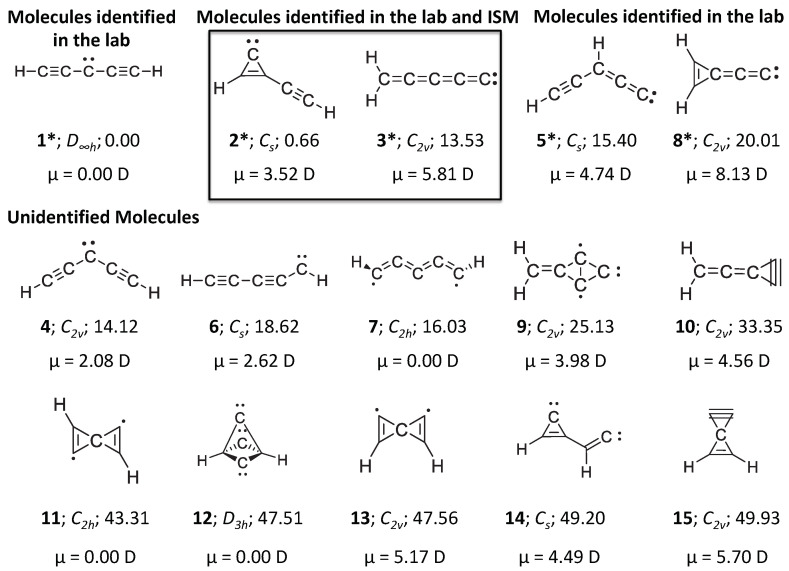
C${}_{5}$H${}_{2}$ isomers considered in this work. ZPVE-corrected relative energies are calculated at the CCSDT(Q)/CBS level of thee theory (in kcal mol${}^{-1}$). Dipole moments (in Debye) and ZPVEs are computed at the CCSD(T)/cc-pVTZ level of the theory. Experimentally detected isomers are marked with an asterisk. Isomer **1** is a triplet and all others are singlets. Note that isomers **10** and **15** are transition states, isomer **7** is a second-order saddle-point, and all others are local minima.

Until now, five C${}_{5}$H${}_{2}$ isomers, namely, linear pentadiynylidene (**1**) [20,21,22,23,24,25,26], ethynylcyclopropenylidene (**2**) [6,23,26], pentatetraenylidene (**3**) [2,21,24], ethynylpropadienylidene (**5**) [21,27], and 2-cyclopropen-1-ylidenethenylidene (**8**) [25,27] have been identified in the laboratory (see Figure 1). Using Fourier Transform Microwave (FTMW) spectroscopy, McCarthy and co-workers had identified four closed-shell carbene isomers of C${}_{5}$H${}_{2}$ (**2**, **3**, **5** and **8**), whose dipole moments are non-zero (μ≠0). Due to symmetry ($D_{\infty h}$), isomer **1** lacks a permanent dipole and, therefore, this molecule is unsuitable for rotational spectroscopic observations and radioastronomical studies. Among the four polar carbenes that were identified in the laboratory, the cumulene carbene isomer (**3**) was reported initially [2]. Later, the laboratory detection of the three-membered ring-chain isomer (**2**) was also reported with both *a*- and *b*-type rotational transitions, as the inertial axis dipole moment components are in two directions for this molecule [6]. Both of these molecules (**3** and **2**) are higher homologues of propadienylidene (a cumulene carbene isomer of C${}_{3}$H${}_{2}$) and cyclopropenylidene, respectively. In addition, the latter are known to exist in the interstellar medium (ISM) [28,29]. The laboratory detection of C${}_{3}$H${}_{2}$ isomers was undoubtedly very helpful in the identification of the same in the ISM [28,30]. Three isotopologues of C${}_{3}$H${}_{2}$, the doubly deuterated c-C${}_{3}$D${}_{2}$, singly deuterated c-C${}_{3}$HD, and one species of c-H${}^{13}$CC${}_{2}$H (${}^{13}$C off of the principal axis of the molecule) have been detected in two starless cores [31,32,33,34]. In 2021, two of the polar C${}_{5}$H${}_{2}$ isomers, **2** and **3**, were identified in the Taurus Molecular Cloud-1 (TMC-1) [35,36]. Though numerous theoretical studies have been carried out in the past, the focus was mostly on the structure, energetics, and spectroscopic properties of the low-lying isomers alone that were observed in the laboratory [3,9,20,21,22,23,24,25,37,38,39,40,41,42,43,44]. A comprehensive study related to the accuracy of rotational constants with various theoretical methods has not been carried out so far and, thus, the objective of the present article focuses on that front.

It should be noted that the electronic structure of the C${}_{5}$H${}_{2}$ isomers in Figure 1 renders these isomers challenging from the electronic structure point of view. As illustrated in Figure 1, these isomers are characterized by carbenes (mono, bi, or tri), biradicals, polyynics, molecules with a planar tetracoordinate carbon (ptC) atom [15,43,45,46,47,48,49] or cumulenic electronic structures [41,42,43]. In addition, several isomers are associated with high strain energies. Therefore, these isomers are expected to be challenging for density functional theory (DFT) methods, and high-level ab initio methods such as coupled-cluster with single, double, and quasiperturbative triple excitations (CCSD(T)) [50,51,52,53] are needed for an adequate description of the electronic structure of these isomers. Having said that, the CCSD(T) method at the complete basis set (CBS) limit has been found to reproduce the relative energies of the C${}_{5}$H${}_{2}$ isomers with sub-chemical accuracy (i.e., with deviations from CCSDT(Q)/CBS energies below 1 kcal mol${}^{-1}$) [41,42,43,54].

## 2. Computational Details

Geometry optimizations and frequency calculations for all C${}_{5}$H${}_{2}$ isomers are carried out with 12 different density functionals with the cc-pVQZ basis set [55]. The considered DFT functionals are: PBE [56], BLYP [57,58], M06-L [59,60], TPSS [61,62], B3LYP [57,58,63,64], PBE0 [65], BMK [66], M06-2X [60], ωB97X-D [67], CAM-B3LYP [68], B2-PLYP [69], and DSD-PBEP86 [70]. To account for London dispersion interactions [71], Grimme’s empirical dispersion corrections (D3) [72] with Becke-Johnson damping (D3BJ) [73,74] has been included in all DFT calculations except M06-L, M06-2X, and ωB97X-D, where dispersion effects are already accounted for. All DFT calculations are carried out with the Gaussian 16 program suite [75]. Apart from using DFT, both geometry optimization and frequency calculations were also performed using coupled-cluster (CC) methods with singles and doubles (CCSD) [76,77] augmented with perturbative treatments of triple excitations (CCSD(T)) to incorporate a high-level treatment of electron correlation effects [50,51,52]. We have used Dunning’s correlation consistent polarized valence double, triple, and quadruple zeta (cc-pV*n*Z; *n* = D, T, and Q) basis sets [55] in these calculations. The frozen-core (fc) approximation is utilized (i.e., the carbon 1s orbitals are frozen) in these calculations. All-electron (ae) CCSD(T) calculations were also carried out, and we have used both core-valence (cc-pCV*n*Z; *n* = T, and Q) [78] and weighted core-valence (cc-pwCVTZ) [79] basis sets in these cases. We note that the fc-CCSD(T)/cc-pVTZ level of the theory has been found to give accurate equilibrium geometries with a mean absolute deviation of only 0.003 Årelative to CCSD(T)/CBS bond distances for a wide and diverse set of the 122 species with up to five non-hydrogen atoms [80]. Harmonic vibrational frequencies were computed at the same levels of the theory by analytic calculation of second derivatives for all stationary points [81]. All CCSD(T) calculations were carried out with the CFOUR program package [82]. For the sake of simplicity, hereinafter, CCSD(T) will be denoted by CC.

## 3. Results and Discussion

Let us begin with the CC results for the molecules that have been synthesized in the laboratory (isomers **2**, **3**, **5**, and **8** shown in Figure 1). Table 1 lists the equilibrium CC rotational constants along with the semi-experimental rotational constants for isomer **2**, **3**, **5**, and **8**. We begin by noting that the deviations between the theory and experiments are very large for the $A_{e}$ rotational constant for the isomers **2** and **8**, both of which contain a highly strained cyclopropene ring. It should be noted, however, that excluding these three problematic rotational constants, the percentage errors obtained for the other isomers at the fc-CC/cc-pVTZ level of the theory are on par with those obtained at the same level of the theory for a set of less challenging small molecules in reference [17]. For the three problematic rotational constants ($A_{e}$ for isomers **2** and **8** and $B_{e}$ for isomer **3**), we obtain very high percentage errors of 6.3–10.4% across the various CC levels of the theory in Table 1. These high percentage errors indicate that the $A_{e}$ rotational constant for these isomers poses a challenge even at the CC level. However, these large deviations between the theory and our semi-experimental values may also be attributed to the B3LYP/jun-cc-pVTZ vibrational corrections used to convert the experimental $A_{0}$ rotational constant to semi-experimental equilibrium $A_{e}$ values that can be compared with the CC equilibrium values. Obtaining the vibrational corrections at higher levels of the theory (e.g., at the CC level) proved beyond our available computational resources.

Let us move to examining the results for the $B_{e}$ and $C_{e}$ rotational constants for isomer **2**. The frozen-core CC/cc-pVDZ level of the theory results in large deviations from the semi-experimental values; namely, we obtain deviations of 139 ($B_{e}$) and 145 ($C_{e}$) MHz. These translate into percentage errors of 4.0 and 4.6% relative to experiment. Moving to a triple-ζ quality basis set reduces these errors by a factor of ∼2–3. Namely, the frozen-core CC/cc-pVTZ level of the theory results in deviations from the experiment of 55 ($B_{e}$) and 66 ($C_{e}$) MHz, which translate to percentage errors of 1.6 and 2.1%. Likewise, moving to a quadruple-ζ quality basis set further reduces these errors by a factor of ∼3–4 relative to the fc-CC/cc-pVDZ level of the theory. Specifically, the frozen-core CC/cc-pVQZ level of the theory results in deviations from the experiment of 40 ($B_{e}$) and 51 ($C_{e}$) MHz. These errors translate into percentage errors that approach the 1% mark, namely, 1.1% ($B_{e}$) and 1.6% ($C_{e}$). Thus, overall the fc-CC/cc-pV*n*Z series (*n* = D, T, Q) exhibits a fairly monotonic basis set convergence.

Let us move to the all-electron CC calculations. Correlating the carbon 1s core electrons does not lead to a significant improvement over the poor performance of the valence CC/cc-pVDZ level of the theory (Table 1). However, correlating the core electrons in conjunction with the CC/cc-pVTZ level of the theory results in a significant improvement over the frozen-core CC/cc-pVTZ level of the theory. Specifically, the deviations between the theory and the experiments are 20 ($B_{e}$) and 34 ($C_{e}$) MHz. These deviations translate to percentage errors that are approaching or below the 1% mark, namely 0.6% ($B_{e}$) and 1.1% ($C_{e}$). However, it should be noted that the cc-pV*n*Z basis sets do not include polarization functions for the core electrons, and were not designed for correlated all-electron calculations. The all-electron CC/cc-pCVTZ level of the theory results in higher percentage errors of 1.2% ($B_{e}$) and ∼1.7% ($C_{e}$) (Table 1). The weighted core-valence cc-pwCVTZ basis set, which provides a better description of core-valence correlation effects, performs slightly better than the older cc-pCVTZ basis set with percentage errors of 1.0% ($B_{e}$) and ∼1.5% ($C_{e}$). The fact that the all-electron CC/cc-pVTZ level of the theory results in better performance than the all-electron CC/cc-pCVTZ and CC/cc-pwCVTZ levels of theory indicates that the former benefits from some degree of fortuitous error cancellations. We note that we were unable to run the all-electron CC/cc-pwCVQZ (or all-electron CC/cc-pVQZ) calculations with the computational resources available to us. However, based on the above results, we expect that the all-electron CC/cc-pwCVQZ level of the theory would provide a further improvement over the all-electron CC/cc-pwCVTZ level of the theory.

The cumulenic isomer **3** has $C_{2v}$ symmetry, and therefore we were able to calculate the all-electron CC rotational constants with the large cc-pCVQZ basis set (Table 1). Before discussing these results, we note that comparisons with the experiment are only made for the $B_{e}$ and $C_{e}$ rotational constants, since direct measurement of the $A_{e}$ rotational constant is not available in the literature [2]. For the $C_{e}$ rotational constant, the CC calculations in conjunction with a double-ζ quality basis set (either in conjunction with the frozen-core approximation or with all-electrons correlated) result in poor rotational constants with percentage errors of ∼3% relative to the experiment. These results are similar to those obtained for the $B_{e}$ and $C_{e}$ rotational constants of isomer **2**. However, for the $B_{e}$ rotational constant of isomer **3**, the CC/cc-pVDZ level of the theory (either in conjunction with the frozen-core approximation or with all-electrons correlated) results in near-zero percentage errors of 0.1–0.3%. This result is likely due to fortuitous cancellations between basis set incompleteness errors and the B3LYP/jun-cc-pVTZ vibrational corrections used to convert the experimental rotational constant to semi-experimental equilibrium values. We note that the CC/cc-pVDZ level of the theory consistently attains poor results for all other cases in Table 1. Apart from this anomaly for the $B_{e}$ rotational constant of isomer **3**, the frozen core CC/cc-pV*n*Z series (*n* = D, T, Q) exhibits a monotonic basis-set convergence, where the percentage errors are consistently reduced when moving from the cc-pVDZ to the cc-pVTZ basis set and from the cc-pVTZ to the cc-pVQZ basis set (Table 1). Correlating all electrons at the all-electron CC/cc-pVTZ level of the theory results in a near-zero percentage error of 0.05% for the $C_{e}$ rotational constant. Again, this exceptionally good agreement with the experiment is likely fortuitous, and as it the case for isomer **2**, the addition of core polarization functions in the all-electron CC/cc-pCVTZ level of the theory results in a noticeably larger percentage error of and 0.5% ($C_{e}$). The weighted core-valence cc-pwCVTZ basis set results in a similar percentage error of 0.4% for the $C_{e}$ rotational constant. For the highly symmetric **3** isomer, we were able to obtain the rotational constants at the all-electron CC/cc-pCVQZ level of the theory, which provides a significant improvement over the all-electron CC/cc-pCVTZ and CC/cc-pwCVTZ results, with percentage errors of 0.06% for the $C_{e}$ rotational constant. We note that for the $B_{e}$ rotational constant of isomer **3**, correlating all the electrons does not provide an improvement over the frozen-core calculations. Again, this anomalous behavior is not observed for the other isomers in Table 1.

For isomer **5**, we obtain monotonic convergence along the frozen core CC/cc-pV*n*Z series (*n* = D, T, Q) for the three rotational constants ($A_{e}$, $B_{e}$, and $C_{e}$). As expected, the CC/cc-pVDZ level of the theory performs poorly, with the percentage errors ranging between 2.8% ($A_{e}$) and 3.3% ($B_{e}$). The CC/cc-pVTZ level of the theory approaches or achieves percentage errors below the 1% mark; namely, the percentage errors range between 0.7% ($A_{e}$) and 1.1% ($B_{e}$). The CC/cc-pVQZ level of the theory performs well, with all percentage errors ≤0.4%. Namely, we obtain percentage errors of 0.31% ($A_{e}$), 0.44% ($B_{e}$), and 0.35% ($C_{e}$). Inspection of Table 1 reveals that correlating all electrons does not lead to an improvement over the frozen-core CC results for isomer **5**.

Isomer **8** has $C_{2v}$ symmetry, and therefore we were able to calculate the all-electron CC rotational constants with the large cc-pCVQZ basis set (Table 1). As mentioned above, the percentage errors for the $A_{e}$ rotational constant are very large, potentially due to the B3LYP/jun-cc-pVTZ vibrational corrections used to convert the experimental $A_{0}$ rotational constant to a semi-experimental equilibrium $A_{e}$ value. For the $B_{e}$ and $C_{e}$ rotational constants, we obtain monotonic convergence along the frozen-core CC/cc-pV*n*Z series (*n* = D, T, Q). The CC/cc-pVQZ level of the theory performs well for the $B_{e}$ rotational constant with a percentage error of 0.2%; however, for the $C_{e}$ rotational constant, a relatively large percentage error of 1.2% is obtained. Correlating all electrons in the CC/cc-pCVQZ calculations results in percentage errors below the 1% mark for both rotational constants; namely, we obtain percentage errors of 0.31% ($B_{e}$) and 0.77% ($C_{e}$).

The above results show that for most of the rotational constants, the fc-CC/cc-pV*n*Z series (*n* = D, T, Q) exhibits a fairly monotonic basis set convergence and that the CC/cc-pVTZ level of the theory (in conjunction with the frozen-core approximation or with all electrons correlated) results in percentage errors approaching or below the 1% mark. However, for a subset of the rotational constants (namely, $A_{e}$ of isomers **2** and **8**, and $B_{e}$ of isomer **3**), very large percentage errors above 3% are obtained, potentially due to the B3LYP/jun-cc-pVTZ vibrational corrections used to convert the experimental rotational constant to semi-experimental equilibrium values that can be compared with the CC results. Additional factors that could affect the above percentage errors are the complete neglect of anharmonicity and post-CC effects, as well as issues associated with inaccurate geometries. To isolate the effects of basis set size and correlating the core electrons at the CC level, it is instructive to compare the fc-CC and ae-CC levels to the best available level of the theory in Table 1. These results are presented in Table 2. The frozen-core CC/cc-pVDZ attains large percentage errors of ∼3% in nearly all cases. Correlating all electrons does not lead to a performance improvement at this level of the theory. The frozen-core CC/cc-pVTZ results in significantly better performance with nearly all percentage errors ≤0.5%. The two exceptions to this are the $A_{e}$ rotational constant of isomer **2** with a percentage error of 0.8% and the $B_{e}$ rotational constant of isomer **5** with a percentage error of 0.6%. Here, correlating all electrons does lead to an improvement in performance, with the notable exception of the $A_{e}$ rotational constant of isomer **5**. Using the cc-pCVTZ or cc-pwCVTZ basis sets in the ae-CC calculations does not lead to an improvement in performance over the cc-pVTZ basis set, indicating that the latter benefits from some degree of fortuitous error cancellation.

We now focus our attention on the comparison of rotational constants calculated using various DFT methods across the rungs of Jacob’s Ladder [83]. We also refer the reader to several related benchmark studies which considered other species [84,85,86,87]. We will primarily compare the DFT results to the best level of the theory in Table 1, namely ae-CC/cc-pwCVTZ for isomers **2** and **5**, and ae-CC/cc-pCVQZ for isomers **3** and **8** (for a comparison between the DFT results and the semi-experimental values, see the Supplementary Materials). The DFT rotational constants, the deviations from our best CC values, and the percentage errors are given in Table 3. In the following discussion, we will focus on functionals that are able to consistently achieve percentage errors from CC below 1% across all isomers (**2**, **3**, **5**, and **8**) and rotational constants ($A_{e}$, $B_{e}$, and $C_{e}$). Let us begin with several general observations. The two range-separated hybrid functionals CAM-B3LYP-D3BJ and ωB97X-D show poor performance and cannot achieve this goal. The global hybrid functionals B3LYP-D3BJ and PBE0-D3 show better performance and result in percentage errors approaching (or below) 1% for the $B_{e}$ and $C_{e}$ rotational constants for all isomers but not for the $A_{e}$ rotational constants. We, therefore, do not recommend the use of these range-separated and global hybrid functionals for the calculation of rotational constants in similar systems. Moving to the hybrid-meta generalized gradient approximation (GGA) methods from rung 4 of Jacob’s Ladder, BMK-D3BJ shows improved performance relative to M06-2X. In particular, BMK-D3BJ results in percentage errors ≤1.0% for all but two rotational constants. The two rotational constants for which the percentage error is 1.3% are $B_{e}$ and $C_{e}$ of isomer **5**. However, as we shall see below, BMK-D3BJ does not provide a significant improvement over the lower-level GGA and meta-GGA methods. The M06-2X method results in percentage errors ≤ 1.0% for only four rotational constants ($A_{e}$ of isomer **3** and all rotational constants of isomer **8**).

Let us move to the pure DFT methods from rungs 2 and 3 of Jacob’s Ladder. Both GGA methods BLYP-D3BJ and PBE-D3BJ result in percentage errors from CC below 1% across nearly all isomers and rotational constants. This is indeed remarkable, considering that the hybrid GGA methods were unable to achieve this goal. In particular, BLYP-D3BJ attains percentage errors ≤0.7% for all but three rotational constants (Table 3). Similarly, PBE-D3BJ attains percentage errors ≤0.7% for all but four rotational constants. Let us move to the performance of the meta-GGA methods, which additionally employ the kinetic energy density. The highly empirical M06-L method results in poor performance with percentage errors ≥1% for half of the rotational constants. However, the non-empirical TPSS meta-GGA functional results in exceptional performance, with practically all percentage errors being below 0.6%. Furthermore, with the exception of two additional rotational constants ($B_{e}$ and $C_{e}$ for isomer **8**), TPSS-D3BJ results in percentage errors being below 0.3%.

Let us move on to the double-hybrid DFT methods, which involve both Hartree–Fock exchange and MP2-like correlation from second-order Møller–Plesset perturbation theory. The older generation B2-PLYP functional shows good performance with nearly all percentage errors below 0.8%. However, it should be noted that overall, the meta-GGA TPSS-D3BJ method provides better performance than B2-PLYP. The DSD-PBEP86-D3BJ functional in which the same-spin and opposite-spin components of the correlation energy are scaled by empirically motivated scaling factors achieves significantly better performance than B2-PLYP. Namely, with the exception of $A_{e}$ for isomer **2** and $B_{e}$ for isomer **5**, all the percentage errors for DSD-PBEP86 are below 0.5%. Furthermore, for half of the rotational constants, the percentage errors are below the 0.2% mark. Together with previous findings [84,85,86,88,89], these results place DSD-PBEP86-D3BJ as an excellent functional for the prediction of geometrical and spectroscopic properties. However, we note that this excellent performance comes with a significant increase in the computational cost. Whereas the computational cost of the TPSS method, which is the second-best performer, scales as ∼$N_{bas}^{3}$ with respect to the number of basis functions, DSD-PBEP86 calculations scale as ∼$N_{bas}^{5}$. Still, DSD-PBEP86 calculations are computationally far more economical than CC calculations, which scale as ∼$N_{bas}^{7}$ [54]. Overall, we would recommend using DSD-PBEP86-D3BJ for relatively small systems, such as those considered in the present work, and TPSS-D3BJ for significantly larger systems.

So far, we have discussed the performance of the isomers **2**, **3**, **5**, and **8**, which have been synthesized in the lab, and for which experimental rotational constants have been measured. We have examined the performance of the CC method in conjunction with a variety of basis sets and treatment of the core electrons, and also identified the DFT methods, which are able to systematically reproduce the best CC rotational constants with percentage errors below the 1% mark. It is of interest to use these accurate CC and DFT methods for calculating the rotational constants of the isomers for which experimental data are not available. These results are given in Table 4. Both isomers **4** and **6** could be considered acetylenic carbenes from the electronic structure point of view, which are well-known intermediates in many prototypical organic reactions [22,90,91]. It is noted here that isomers **4**, **6**, and **9** may have been overlooked in previous experimental and theoretical studies [2,3,21]. On the contrary, isomers **11** and **13** have been considered in previous theoretical studies, as they contain the unconventional planar tetracoordinate carbon atoms [92,93]. Nevertheless, the rotational constants have not been reported in these studies, and the focus was rather related to the chemical bonding aspects of these molecules. It is noted here that **7** is a second-order saddle point, whereas **10** and **15** are transition states. It is also noted here that isomers **11** and **12** lack permanent dipole moments due to symmetry, and thus they are not suitable candidates for FTMW spectroscopy. We note that the geometry of **4** rearranges to **6** at the fc-CC/cc-pVDZ level of the theory, indicating that cc-pVDZ basis set is not adequate for these fluxional molecules. Also, for **4**, the $A_{e}$ rotational constant value obtained at the TPSS-D3BJ/cc-pVQZ level seems to be problematic, as the equilibrium geometry nearly rearranges to **6**. In addition, although the $B_{e}$ and $C_{e}$ rotational constants are consistent between DSD-PBEP86 and ae-CC methods for **6**, the $A_{e}$ rotational constant value is inconsistent between these methods. Therefore, unless these molecules are experimentally identified in the laboratory and rotational constants are measured, it is difficult to reach a conclusion as far as which method is most accurate. However, such issues seem to disappear for isomers **9** and **13**, as they are not fluxional molecules like **4** and **6**. Therefore, for **9** and **13** one could rely on the values obtained from ae-CC/cc-pVQZ and DSD-PBEP86/cc-pVQZ levels.

## 4. Conclusions

This work considers a series of challenging carbene C${}_{5}$H${}_{2}$ isomers with cumulene, acetylene, or strained cyclopropene moieties to examine the theoretical rotational constants predicted by frozen-core and all-electron CCSD(T) calculations, as well as a range of DFT methods. The considered C${}_{5}$H${}_{2}$ isomers (**2**, **3**, **5**, and **8**) have been experimentally identified using FTMW spectroscopy, and two of them (**2** and **3**) have been recently confirmed in the interstellar medium. The calculated rotational constants are compared to semi-experimental rotational constants obtained by converting the vibrationally averaged experimental rotational constants ($A_{0}$, $B_{0}$, and $C_{0}$) to equilibrium values by subtracting the vibrational contributions (calculated at the B3LYP/jun-cc-pVTZ level of the theory). We find that calculating the equilibrium rotational constants of these C${}_{5}$H${}_{2}$ isomers is a difficult task, even at the CCSD(T) level. For example, at the all-electron CCSD(T)/cc-pwCVTZ level of the theory, we obtain percentage errors ≤0.4% ($C_{e}$ of isomer **3**, $B_{e}$ and $C_{e}$ of isomer **5**, and $B_{e}$ of isomer **8**) and 0.9–1.5% ($B_{e}$ and $C_{e}$ of isomer **2**, $A_{e}$ of isomer **5**, and $C_{e}$ of isomer **8**), whereas for the $A_{e}$ rotational constant of isomers **2** and **8** and $B_{e}$ rotational constant of isomer **3**, high percentage errors above 3% are obtained. These results highlight the challenges associated with calculating accurate rotational constants for isomers with highly challenging electronic structures, which is further complicated by the need to convert vibrationally averaged experimental rotational constants to equilibrium values. We use our best CCSD(T) rotational constants (namely, ae-CCSD(T)/cc-pwCVTZ for isomers **2** and **5**, and ae-CCSD(T)/cc-pCVQZ for isomers **3** and **8**) to evaluate the performance of DFT methods across the rungs of Jacob’s Ladder. We find that the considered pure functionals (BLYP-D3BJ, PBE-D3BJ, and TPSS-D3BJ) perform significantly better than the global and range-separated hybrid functionals. The double-hybrid DSD-PBEP86-D3BJ method shows the best overall performance with percentage errors below 0.5% in nearly all cases. Therefore, we recommend the use of the DSD-PBEP86-D3BJ method for relatively small systems, whereas for larger systems, the TPSS-D3BJ method is recommended. We hope that these results will help identify the unknown isomers (**4**, **6**, **9**, **13**) in the laboratory and, consequently, in the interstellar medium.

## Figures and Tables

**Table 1:** Theoretical equilibrium rotational constants ($A_{e}$, $B_{e}$, $C_{e}$; in MHz) obtained at the CCSD(T) level using different basis sets (with and without the frozen-core approximation) along with semi-experimental rotational constants for isomers **2**, **3**, **5**, and **8** of C${}_{5}$H${}_{2}$${}^{a}$.

		Rotational Constants			% Error		
**Isomer**	**Level of the Theory ${}^{b}$**	$A_{e}$	$B_{e}$	$C_{e}$	$A_{e}$	$B_{e}$	$C_{e}$
**2**	Semi-Experimental ${}^{c}$	36,972.92	3448.64	3154.31	-	-	-
	fc-CC/VDZ	33,109.37	3309.59	3008.83	−10.45 ${}^{g}$	−4.03	−4.61
	fc-CC/VTZ	34,266.70	3394.14	3088.25	−7.32 ${}^{g}$	−1.58	−2.09
	fc-CC/VQZ	34,547.62	3409.12	3102.93	−6.56 ${}^{g}$	−1.15	−1.63
	ae-CC/VDZ	33,184.61	3315.35	3014.21	−10.25 ${}^{g}$	−3.87	−4.44
	ae-CC/VTZ	34,640.92	3429.06	3120.20	−6.31 ${}^{g}$	−0.57	−1.08
	ae-CC/CVTZ	34,465.55	3408.64	3101.86	−6.78 ${}^{g}$	−1.16	−1.66
	ae-CC/CVTZ	34,544.69	3414.01	3106.95	−6.57 ${}^{g}$	−1.00	−1.50
**3**	Semi-Experimental ${}^{d}$	231,551.05 ^e^	2216.88	2283.64	-	-	-
	fc-CC/VDZ	282,533.83	2219.13	2201.84	N/A	0.10	−3.58
	fc-CC/VTZ	289,957.56	2280.08	2262.29	N/A	2.85	−0.93
	fc-CC/VQZ	290,263.96	2289.74	2271.82	N/A	3.29	−0.52
	ae-CC/VDZ	283,066.03	2223.09	2205.77	N/A	0.28	−3.41
	ae-CC/VTZ	292,400.34	2302.87	2284.87	N/A	3.88	0.05
	ae-CC/CVTZ	290,592.49	2289.69	2271.79	N/A	3.28	−0.52
	ae-CC/wCVTZ	290,836.71	2293.17	2275.23	N/A	3.44	−0.37
	ae-CC/CVQZ	291,115.51	2300.31	2282.27	N/A	3.76	−0.06
**5**	Semi-Experimental ${}^{f}$	31,151.23	2863.13	2621.54	-	-	-
	fc-CC/VDZ	30,282.06	2769.12	2537.12	−2.79	−3.28	−3.22
	fc-CC/VTZ	31,364.94	2832.59	2597.97	0.69	−1.07	−0.90
	fc-CC/VQZ	31,248.01	2850.63	2612.32	0.31	−0.44	−0.35
	ae-CC/VDZ	30,321.18	2774.08	2541.55	−2.66	−3.11	−3.05
	ae-CC/VTZ	32,467.69	2842.48	2613.66	4.23	−0.72	−0.30
	ae-CC/CVTZ	31,402.16	2846.75	2610.13	0.81	−0.57	−0.44
	ae-CC/wCVTZ	31,431.40	2851.69	2614.48	0.90	−0.40	−0.27
**8**	Semi-Experimental ${}^{f}$	34,494.31	3515.75	3202.60	-	-	-
	fc-CC/VDZ	31,020.01	3398.87	3063.23	−10.07 ${}^{g}$	−3.32	−4.35
	fc-CC/VTZ	31,836.35	3492.11	3146.92	−7.71 ${}^{g}$	−0.67	−1.74
	fc-CC/VQZ	31,978.63	3509.58	3162.50	−7.29 ${}^{g}$	−0.18	−1.25
	ae-CC/VDZ	31,070.60	3405.23	3068.89	−9.93 ${}^{g}$	−3.14	−4.18
	ae-CC/VTZ	32,080.69	3528.59	3178.94	−7.00 ${}^{g}$	0.37	−0.74
	ae-CC/CVTZ	31,979.91	3508.69	3161.79	−7.29 ${}^{g}$	−0.20	−1.27
	ae-CC/wCVTZ	32,036.11	3514.77	3167.28	−7.13 ${}^{g}$	−0.03	−1.10
	ae-CC/CVQZ	32,122.26	3526.79	3177.88	−6.88 ${}^{g}$	0.31	−0.77

^*a*^ Experimental $A_{0}$, $B_{0}$, and $C_{0}$ values are converted to semi-experimental $A_{e}$, $B_{e}$, and $C_{e}$ values by subtracting the vibrational contributions calculated at the B3LYP/jun-cc-pVTZ level of the theory. ^*b*^ For simplicity, V*n*Z indicates cc-pV*n*Z, CV*n*Z indicates cc-pCV*n*Z, and wCV*n*Z indicates cc-pwCV*n*Z. ^*c*^ Experimental $A_{0}$, $B_{0}$, and $C_{0}$ values from reference [6]. ^*d*^ Experimental $A_{0}$, $B_{0}$, and $C_{0}$ values from reference [2]. ^*e*^ This is not a measured value. This value has been derived assuming a planar structure, that is, 1/C − 1/A − 1/B = 0. ^*f*^ Experimental $A_{0}$, $B_{0}$, and $C_{0}$ values from reference [27]. ^*g*^ We note that the high percentage errors for $A_{e}$ of isomers **2** and **8** are partially attributed to the B3LYP/jun-cc-pVTZ vibrational corrections used to convert the experimental $A_{0}$ rotational constant to semi-experimental equilibrium $A_{e}$ value that can be compared with the CC equilibrium values.

**Table 2:** Percentage errors in theoretical equilibrium rotational constants ($A_{e}$, $B_{e}$, $C_{e}$) for isomers **2**, **3**, **5**, and **8** obtained at the CC level using different basis sets (with and without the frozen-core approximation) relative to the highest level of the theory in Table 1.

		% Error		
**Isomer**	**Level of the Theory ${}^{a}$**	$A_{e}$	$B_{e}$	$C_{e}$
**2**	fc-CC/VDZ ${}^{b}$	−4.16	−2.92	−3.03
	fc-CC/VTZ ${}^{b}$	−0.81	−0.44	−0.47
	ae-CC/VDZ ${}^{c}$	−3.94	−2.89	−2.98
	ae-CC/VTZ ${}^{c}$	0.28	0.44	0.43
	ae-CC/CVTZ ${}^{c}$	−0.23	−0.16	−0.16
**3**	fc-CC/VDZ ${}^{b}$	−2.66	−3.08	−3.08
	fc-CC/VTZ ${}^{b}$	−0.11	−0.42	−0.42
	ae-CC/VDZ ${}^{d}$	−2.77	−3.36	−3.35
	ae-CC/VTZ ${}^{d}$	0.44	0.11	0.11
	ae-CC/CVTZ ${}^{d}$	−0.18	−0.46	−0.46
	ae-CC/wCVTZ ${}^{d}$	−0.10	−0.31	−0.31
**5**	fc-CC/VDZ ${}^{b}$	−3.09	−2.86	−2.88
	fc-CC/VTZ ${}^{b}$	0.37	−0.63	−0.55
	ae-CC/VDZ ${}^{c}$	−3.53	−2.72	−2.79
	ae-CC/VTZ ${}^{c}$	3.30	−0.32	−0.03
	ae-CC/CVTZ ${}^{c}$	−0.09	−0.17	−0.17
**8**	fc-CC/VDZ ${}^{b}$	−3.00	−3.15	−3.14
	fc-CC/VTZ ${}^{b}$	−0.44	−0.50	−0.49
	ae-CC/VDZ ${}^{d}$	−3.27	−3.45	−3.43
	ae-CC/VTZ ${}^{d}$	−0.13	0.05	0.03
	ae-CC/CVTZ ${}^{d}$	−0.44	−0.51	−0.51
	ae-CC/wCVTZ ${}^{d}$	−0.27	−0.34	−0.33

^*a*^ For simplicity, V*n*Z indicates cc-pV*n*Z, CV*n*Z indicates cc-pCV*n*Z, and wCV*n*Z indicates cc-pwCV*n*Z. ^*b*^ Percentage errors are calculated relative to the fc-CC/cc-pVQZ level of the theory. ^*c*^ Percentage errors are calculated relative to the ae-CC/cc-pwCVTZ level of the theory. ^*d*^ Percentage errors are calculated relative to the ae-CC/cc-pCVQZ level of the theory.

**Table 3:** Theoretical equilibrium rotational constants ($A_{e}$, $B_{e}$, $C_{e}$; in MHz) obtained using DFT methods across the rungs of Jacob’s Ladder. These results are compared to the best CC results from Table 1 for Isomers **2**, **3**, **5**, and **8**.

		Rotational Constants			% Error		
**Isomer**	**Level of the Theory**	$A_{e}$	$B_{e}$	$C_{e}$	$A_{e}$	$B_{e}$	$C_{e}$
**2** ${}^{a}$	PBE-D3BJ	34,776.97	3410.3	3105.75	0.67	−0.11	−0.04
	BLYP-D3BJ	34,772.95	3406.65	3102.68	0.66	−0.22	−0.14
	TPSS-D3BJ	34,832.08	3421.18	3115.2	0.83	0.21	0.27
	BMK-D3BJ	34,305.88	3417.45	3107.86	−0.69	0.10	0.03
	B2-PLYP-D3BJ	35,080.47	3437.28	3130.54	1.55	0.68	0.76
	DSD-PBEP86-D3BJ	34,906.49	3425.77	3119.61	1.05	0.34	0.41
**3** ${}^{b}$	PBE-D3BJ	288,084.24	2289.71	2271.66	−1.04	−0.46	−0.46
	BLYP-D3BJ	290,215.02	2292.37	2274.41	−0.31	−0.35	−0.34
	TPSS-D3BJ	291,046.32	2299.95	2281.92	−0.02	−0.02	−0.02
	BMK-D3BJ	290,741.94	2324.26	2305.82	−0.13	1.04	1.03
	B2-PLYP-D3BJ	293,012.38	2313.86	2295.74	0.65	0.59	0.59
	DSD-PBEP86-D3BJ	291,090.37	2305.76	2287.64	−0.01	0.24	0.24
**5** ${}^{a}$	PBE-D3BJ	31,828.92	2844.85	2611.44	1.26	−0.24	−0.12
	BLYP-D3BJ	31,858.95	2844.08	2610.99	1.36	−0.27	−0.13
	TPSS-D3BJ	31,747.91	2858.87	2622.7	1.01	0.25	0.31
	BMK-D3BJ	31,631.84	2889.51	2647.65	0.64	1.33	1.27
	B2-PLYP-D3BJ	31,903.2	2873.29	2635.89	1.50	0.76	0.82
	DSD-PBEP86-D3BJ	31,573.48	2867.58	2628.82	0.45	0.56	0.55
**8** ${}^{b}$	PBE-D3BJ	31,895.1	3495.39	3150.17	−0.71	−0.89	−0.87
	BLYP-D3BJ	31,951.11	3492.37	3148.26	−0.53	−0.98	−0.93
	TPSS-D3BJ	32,043.94	3507.76	3161.66	−0.24	−0.54	−0.51
	BMK-D3BJ	31,945.51	3514.9	3166.5	−0.55	−0.34	−0.36
	B2-PLYP-D3BJ	32,223.32	3538.94	3188.73	0.31	0.34	0.34
	DSD-PBEP86-D3BJ	32,086.32	3531.32	3181.2	−0.11	0.13	0.10

^*a*^ Percentage errors are calculated relative to the ae-CC/cc-pwCVTZ level of the theory. ^*b*^ Percentage errors are calculated relative to the ae-CC/cc-pCVQZ level of the theory.

**Table 4:** Theoretically calculated rotational constants (in MHz) of unknown C${}_{5}$H${}_{2}$ isomers at different CC and DFT levels.

Isomer	Level of the Theory ${}^{a}$	$A_{e}$	$B_{e}$	$C_{e}$
**4**	fc-CC/VTZ	44,468.31	2675.36	2523.54
	fc-CC/VQZ	44,559.66	2687.42	2534.56
	ae-CC/VTZ	47,663.99	2670.19	2528.54
	ae-CC/CVTZ	44,964.29	2683.13	2532.04
	ae-CC/wCVTZ	45,248.67	2684.92	2534.53
	ae-CC/CVQZ	45,285.14	2693.43	2542.23
	TPSS-D3BJ/VQZ	415,092.95	2314.94	2302.10
	DSD-PBEP86-D3BJ/VQZ	48,334.30	2669.57	2529.84
**6**	fc-CC/VTZ	575,100.98	2265.22	2256.33
	fc-CC/VQZ	599,411.76	2273.66	2265.06
	ae-CC/VTZ	611,557.40	2287.33	2278.81
	ae-CC/CVTZ	572,323.22	2274.94	2265.93
	ae-CC/wCVTZ	526,553.84	2282.03	2272.19
	TPSS-D3BJ/VQZ	413,454.77	2315.19	2302.29
	DSD-PBEP86-D3BJ/VQZ	672,216.98	2287.43	2279.67
**9**	fc-CC/VTZ	31,658.89	4569.62	3993.24
	fc-CC/VQZ	31,908.98	4592.16	4014.43
	ae-CC/VTZ	32,178.66	4612.32	4034.09
	ae-CC/CVTZ	31,919.04	4589.89	4012.85
	ae-CC/wCVTZ	32,008.12	4598.06	4020.50
	ae-CC/CVQZ	32,169.05	4612.09	4033.77
	TPSS-D3BJ/VQZ	32,428.41	4572.42	4007.38
	DSD-PBEP86-D3BJ/VQZ	32,191.22	4616.16	4037.23
**13**	fc-CC/VTZ	18,897.05	5445.32	4227.22
	fc-CC/VQZ	19,024.44	5480.23	4254.63
	ae-CC/VTZ	19,101.44	5510.40	4276.67
	ae-CC/CVTZ	19,000.57	5475.68	4250.69
	ae-CC/wCVTZ	19,043.27	5484.32	4258.03
	ae-CC/CVQZ	19,130.16	5509.91	4277.81
	TPSS-D3BJ/VQZ	19,008.80	5482.72	4255.35
	DSD-PBEP86-D3BJ/VQZ	19,144.14	5535.55	4293.95

^*a*^ For simplicity, V*n*Z indicates cc-pV*n*Z, CV*n*Z indicates cc-pCV*n*Z, and wCV*n*Z indicates cc-pwCV*n*Z.

## Supplementary Materials

The following supporting information can be downloaded at: https://www.mdpi.com/article/10.3390/molecules28186537/s1.

## Abbreviations

The following abbreviations are used in this manuscript:

DFT	Density functional theory
CC	CCSD(T), coupled-cluster singles, doubles including perturbative triples
ptC	Planar tetracoordinate carbon
