# Supplementary material for: CCSD(T) Rotational Constants for Highly Challenging C5H2 Isomers—A Comparison between Theory and Experiment
Source: Molecules. 2023 Sep 9;28(18):6537. doi: 10.3390/molecules28186537 (PMC10537648; doi:10.3390/molecules28186537)
Supplement: Supplementary file 1 [file molecules-28-06537-s001.zip › molecules-2558595-supplementary.pdf]

Supporting information for:

CCSD(T) Rotational Constants for Highly Challenging C5H2 Isomers—A Comparison between Theory and Experiment

Venkatesan S. Thimmakondu 1,* and Amir Karton 2,*

1 Department of Chemistry and Biochemistry, San Diego State University, San Diego, CA 92182-1030, USA

2 School of Science and Technology, University of New England, Armidale, NSW 2351, Australia

* Correspondence: vthimmakondusamy@sdsu.edu (V.S.T.); amir.karton@une.edu.au (A.K.)

Contents

List of Figures

List of Tables

S1 Optimized geometries of the C_5H_2 (**1-4**) in their ground electronic states in Cartesian coordinates (in Ångström units) obtained at the fc-CCSD(T)/cc-pVTZ level of theory. S5

S2 Optimized geometries of the C_5H_2 (**5-8**) in their ground electronic states in Cartesian coordinates (in Ångström units) obtained at the fc-CCSD(T)/cc-pVTZ level of theory. S6

S3 Optimized geometries of the C_5H_2 (**9-12**) in their ground electronic states in Cartesian coordinates (in Ångström units) obtained at the fc-CCSD(T)/cc-pVTZ level of theory. S7

S4 Optimized geometries of the C_5H_2 (**13-15**) in their ground electronic states in Cartesian coordinates (in Ångström units) obtained at the fc-CCSD(T)/cc-pVTZ level of theory. S8

S5 Computed energies of C_5H_2 isomers in their ground electronic states calculated at RHF-fc-CCSD(T)/cc-pVTZ level of theory. ZPVEs are scaled by a factor of 0.9868. . . . . S9

S6 Harmonic vibrational frequencies (cm^{-1}) and IR intensities (km mol^{-1}) of the triplet ground electronic state ($\widetilde{X}^3\Sigma_g^-$) of isomer **1** of C_5H_2 calculated at the fc-ROCCSD(T)/cc-pVTZ level of theory. S10

S7 Harmonic vibrational frequencies (cm^{-1}) and IR intensities (km mol^{-1}) of isomer **2** of C_5H_2 calculated at the fc-CCSD(T)/cc-pVTZ level of theory. . . S11

S8 Harmonic vibrational frequencies (cm^{-1}) and IR intensities (km mol^{-1}) of isomer **3** of C_5H_2 calculated at the fc-CCSD(T)/cc-pVTZ level of theory. . . S12

S9 Harmonic vibrational frequencies (cm^{-1}) and IR intensities (km mol^{-1}) of isomer **4** of C_5H_2 calculated at the fc-CCSD(T)/cc-pVTZ level of theory. . . S13

S10 Harmonic vibrational frequencies (cm^{-1}) and IR intensities (km mol^{-1}) of isomer **5** of C_5H_2 calculated at the fc-CCSD(T)/cc-pVTZ level of theory. . . S14

S11 Harmonic vibrational frequencies (cm^{-1}) and IR intensities (km mol^{-1}) of isomer **6** of C_5H_2 calculated at the fc-CCSD(T)/cc-pVTZ level of theory. . . S15

S12 Harmonic vibrational frequencies (cm^{-1}) and IR intensities (km mol^{-1}) of isomer **7** of C_5H_2 calculated at the fc-CCSD(T)/cc-pVTZ level of theory. . . S16

S13 Harmonic vibrational frequencies (cm^{-1}) and IR intensities (km mol^{-1}) of isomer **8** of C_5H_2 calculated at the fc-CCSD(T)/cc-pVTZ level of theory. . . S17

S14 Harmonic vibrational frequencies (cm^{-1}) and IR intensities (km mol^{-1}) of isomer **9** of C_5H_2 calculated at the fc-CCSD(T)/cc-pVTZ level of theory. . . S18

S15 Harmonic vibrational frequencies (cm^{-1}) and IR intensities (km mol^{-1}) of isomer **10** of C_5H_2 calculated at the fc-CCSD(T)/cc-pVTZ level of theory. . S19

S16 Harmonic vibrational frequencies (cm^{-1}) and IR intensities (km mol^{-1}) of isomer **11** of C_5H_2 calculated at the fc-CCSD(T)/cc-pVTZ level of theory. . S20

S17 Harmonic vibrational frequencies (cm^{-1}) and IR intensities (km mol^{-1}) of isomer **12** of C_5H_2 calculated at the fc-CCSD(T)/cc-pVTZ level of theory. . S21

S18 Harmonic vibrational frequencies (cm^{-1}) and IR intensities (km mol^{-1}) of isomer **13** of C_5H_2 calculated at the fc-CCSD(T)/cc-pVTZ level of theory. . S22

S19 Harmonic vibrational frequencies (cm^{-1}) and IR intensities (km mol^{-1}) of isomer **14** of C_5H_2 calculated at the fc-CCSD(T)/cc-pVTZ level of theory. . S23

S20 Harmonic vibrational frequencies (cm^{-1}) and IR intensities (km mol^{-1}) of isomer **15** of C_5H_2 calculated at the fc-CCSD(T)/cc-pVTZ level of theory. . S24

S21 Rotational and centrifugal distortion constants (in MHz), and inertial axis dipole moment components (in Debye; $\perp^r$ representation) of isomers **13** and **14** of C_5H_2 calculated at the fc-CCSD(T)/cc-pVTZ level of theory. S25
S22 Theoretical Equilibrium Rotational Constants (A_e, B_e, C_e; in MHz) Obtained at the CCSD(T) Level Using Different Basis Sets (Obtained With and Without the Frozen-Core Approximation) Along With Experimentally Measured Rotational Constants (A_0, B_0, C_0; in MHz) for Isomers **2**, **3**, **5** of C_5H_2 (see next Table for isomer **8**) S26
S23 Theoretical Equilibrium Rotational Constants (A_e, B_e, C_e; in MHz) Obtained at the CCSD(T) Level Using Different Basis Sets (Obtained With and Without the Frozen-Core Approximation) Along With Experimentally Measured Rotational Constants (A_0, B_0, C_0; in MHz) for Isomer **8** of C_5H_2 . . . . S27

Table S1: Optimized geometries of the C_5H_2 (**1-4**) in their ground electronic states in Cartesian coordinates (in Ångström units) obtained at the fc-CCSD(T)/cc-pVTZ level of theory.

pentadiynylidene (**1**); $\widetilde{X}^3\Sigma_g^-$; $D_{\infty h}$			
C	0.000000	0.000000	0.000000
C	0.000000	0.000000	-1.314136
C	0.000000	0.000000	1.314136
C	0.000000	0.000000	-2.558916
C	0.000000	0.000000	2.558916
H	0.000000	0.000000	-3.622994
H	0.000000	0.000000	3.622994
ethynylcyclopropenylidene (**2**); $\widetilde{X}^1A'$; C_s			
C	-0.348135	0.007666	0.000000
C	1.054041	-0.008613	0.000000
C	2.267753	-0.004875	0.000000
H	3.331810	-0.000758	0.000000
C	-1.565361	0.779726	0.000000
C	-1.516434	-0.641048	0.000000
H	-2.044256	-1.581135	0.000000
pentatetraenylidene (**3**); $\widetilde{X}^1A_1$; C_{2v}			
C	0.000000	0.000000	-1.175733
C	0.000000	0.000000	-2.503913
C	0.000000	0.000000	0.096131
H	0.000000	0.929895	-3.062784
H	0.000000	-0.929895	-3.062784
C	0.000000	0.000000	1.401948
C	0.000000	0.000000	2.696024
bent-pentadiynylidene (**4**); $\widetilde{X}^1A_1$; C_{2v}			
C	0.000000	-1.198855	-0.069317
C	0.000000	0.000000	-0.740830
C	0.000000	1.198855	-0.069317
C	0.000000	-2.348011	0.370524
C	0.000000	2.348011	0.370524
H	0.000000	-3.310583	0.824052
H	0.000000	3.310583	0.824052

Table S2: Optimized geometries of the C_5H_2 (**5**-**8**) in their ground electronic states in Cartesian coordinates (in Ångström units) obtained at the fc-CCSD(T)/cc-pVTZ level of theory.

ethynylpropadienylidene (**5**); $\widetilde{X}^1A'$; C_s			
C	0.082927	-0.726052	0.000000
H	0.078053	-1.814239	0.000000
C	1.253774	-0.064853	0.000000
C	2.401750	0.528349	0.000000
C	-1.185263	-0.085879	0.000000
C	-2.287378	0.425661	0.000000
H	-3.243013	0.894715	0.000000
buta-1,3-diynylcarbene (**6**); $\widetilde{X}^1A'$; C_s			
H	-3.201132	0.831831	0.000000
C	-2.601446	-0.080961	0.000000
C	-1.278515	0.053054	0.000000
C	-0.023219	-0.068528	0.000000
C	1.321958	-0.004486	0.000000
C	2.546870	0.025338	0.000000
H	3.610147	0.068135	0.000000
1,5-cumulenebiradical form of C_5H_2 (**7**); $\widetilde{X}^1A_g$; C_{2h}			
C	0.000000	0.000000	0.000000
C	1.300794	-0.053056	0.000000
C	-1.300794	0.053056	0.000000
C	2.559432	0.083591	0.000000
C	-2.559432	-0.083591	0.000000
H	3.461384	-0.498546	0.000000
H	-3.461384	0.498546	0.000000
2-cyclopropen-1-ylidenethenylidene (**8**); $\widetilde{X}^1A_1$; C_{2v}			
C	0.000000	0.000000	2.392509
C	0.000000	0.000000	1.117825
C	0.000000	0.000000	-0.221601
C	0.000000	-0.669879	-1.473657
C	0.000000	0.669879	-1.473657
H	0.000000	-1.591371	-2.032596
H	0.000000	1.591371	-2.032596

Table S3: Optimized geometries of the C_5H_2 (**9-12**) in their ground electronic states in Cartesian coordinates (in Ångström units) obtained at the fc-CCSD(T)/cc-pVTZ level of theory.

2-methylenebicyclo[1.1.0]but-1(3)-en-4-ylidene (**9**); $\widetilde{X}^1A_1$; C_{2v}			
C	0.000000	0.000000	-0.625215
C	0.000000	0.000000	-1.953631
C	0.000000	-0.769231	0.597650
C	0.000000	0.769231	0.597650
C	0.000000	0.000000	1.802545
H	0.000000	0.934979	-2.494472
H	0.000000	-0.934979	-2.494472
vinylidenecycloprop-1-yne (**10**); $\widetilde{X}^1A_1$; C_{2v}			
C	0.000000	0.000000	2.257558
C	0.000000	0.000000	0.940951
C	0.000000	0.000000	-0.363926
C	0.000000	-0.678576	-1.653737
C	0.000000	0.678576	-1.653737
H	0.000000	0.931120	2.815307
H	0.000000	-0.931120	2.815307
(SP-4)-spiro[2.2]pent-1,4-dien-1,4-diyl (**11**); $\widetilde{X}^1A_g$; C_{2h}			
C	0.000000	0.000000	0.000000
C	1.258597	0.624555	0.000000
C	-1.258597	-0.624555	0.000000
H	1.567862	1.655327	0.000000
H	-1.567862	-1.655327	0.000000
C	1.438668	-0.697891	0.000000
C	-1.438668	0.697891	0.000000
pentacyclo[2.1.0.0^{1,3}.0^{2,5}.0^{3,5}]pentane (**12**); $\widetilde{X}^1A_1'$; D_{3h}			
C	-0.915030	0.000000	0.528293
C	0.000000	0.000000	-1.056586
C	0.915030	0.000000	0.528293
C	0.000000	-1.048972	0.000000
C	0.000000	1.048972	0.000000
H	0.000000	-2.122011	0.000000
H	0.000000	2.122011	0.000000

Table S4: Optimized geometries of the C_5H_2 (**13-15**) in their ground electronic states in Cartesian coordinates (in Ångström units) obtained at the fc-CCSD(T)/cc-pVTZ level of theory.

(SP-4)-spiro[2.2]pent-1,4-dien-1,5-diyl (**13**); $\widetilde{X}^1A_1$; C_{2v}			
C	0.000000	0.000000	0.061467
C	0.000000	-1.375953	-0.748772
C	0.000000	1.375953	-0.748772
C	0.000000	-1.312249	0.585597
C	0.000000	1.312249	0.585597
H	0.000000	-1.731610	1.576956
H	0.000000	1.731610	1.576956
bicarbene (**14**); $\widetilde{X}^1A'$; C_s			
C	-1.121593	-0.498526	0.000000
C	0.279310	-0.125529	0.000000
C	-2.065747	0.424027	0.000000
H	-1.446017	-1.539061	0.000000
C	1.566213	-0.479057	0.000000
C	1.268627	0.913453	0.000000
H	2.317471	-1.251512	0.000000
(SP-4)-spiro[2.2]pent-1-en-4-yne (**15**); $\widetilde{X}^1A_1$; C_{2v}			
C	0.000000	0.000000	-0.013929
C	0.000000	-0.660353	1.269899
C	0.000000	-0.660935	-1.412030
C	0.000000	0.660353	1.269899
C	0.000000	0.660935	-1.412030
H	0.000000	-1.611871	1.775253
H	0.000000	1.611871	1.775253

Table S5: Computed energies of C_5H_2 isomers in their ground electronic states calculated at RHF-fc-CCSD(T)/cc-pVTZ level of theory. ZPVEs are scaled by a factor of 0.9868.

Isomer	Point Group	E a.u	ZPVE[e] kcal mol^{-1}	E+ZPVE a.u	μ Debye	ΔE kcal mol^{-1}	ΔE+ZPVE kcal mol^{-1}
**1**[a]	$D_{\infty h}$	-191.13876266	22.7513	-191.10250619	-	0.0000	0.0000
**2**	C_s	-191.14181701	25.7176	-191.10083344	3.5171	-1.9166	1.0497
**3**	C_{2v}	-191.12155808	25.1024	-191.08155490	5.8052	10.7960	13.1471
**4**	C_{2v}	-191.11584011	23.8331	-191.07785968	2.0825	14.3841	15.4659
**5**	C_s	-191.11884515	25.2072	-191.07867496	4.7439	12.4984	14.9543
**6**	C_s	-191.11225559	23.1723	-191.07532818	2.6159	16.6334	17.0545
**7**[c]	C_{2h}	-191.10885401	22.4023	-191.07315370	-	18.7680	18.4190
**8**	C_{2v}	-191.11168476	25.9934	-191.07026168	8.1297	16.9916	20.2337
**9**	C_{2v}	-191.10510656	25.8528	-191.06390754	3.9795	21.1195	24.2210
**10**[b]	C_{2v}	-191.08813420	24.2753	-191.04944908	4.5629	31.7698	33.2938
**11**	C_{2h}	-191.07321125	24.2880	-191.03450589	-	41.1341	42.6708
**12**	D_{3h}	-191.07009916	26.2104	-191.02833027	-	43.0870	46.5461
**13**	C_{2v}	-191.06623039	24.1759	-191.02770368	5.1731	45.5147	46.9393
**14**	C_s	-191.06476067	24.6738	-191.02544050	4.4904	46.4369	48.3595
**15**[b]	C_{2v}	-191.06150708	24.3100	-191.02276666	5.7010	48.4786	50.0373

[a] For isomer **1** the calculation is done using a ROHF wave function since the electronic state is a triplet. [b] Transition state. [c] Second-order saddle-point.

Table S6: Harmonic vibrational frequencies (cm^{-1}) and IR intensities ($km\ mol^{-1}$) of the triplet ground electronic state ($\widetilde{X}^3\Sigma_g^-$) of isomer **1** of C_5H_2 calculated at the fc-ROCCSD(T)/cc-pVTZ level of theory.

Mode	symmetry	frequency	intensity
1	π_u	113.6	4.8
2	π_u	113.6	4.8
3	π_u	371.7	29.8
4	π_u	371.7	29.8
5	π_g	392.6	0.0
6	π_g	392.6	0.0
7	π_g	420.4	0.0
8	π_g	420.4	0.0
9	π_u	440.3	58.9
10	π_u	440.3	58.9
11	σ_g^+	745.1	0.0
12	σ_u^-	1518.3	10.2
13	σ_u^-	1584.3	4.6
14	σ_g^+	1921.1	0.0
15	σ_u^-	3435.7	188.4
16	σ_g^+	3445.9	0.0

Table S7: Harmonic vibrational frequencies (cm^{-1}) and IR intensities ($km\ mol^{-1}$) of isomer **2** of C_5H_2 calculated at the fc-CCSD(T)/cc-pVTZ level of theory.

Mode	symmetry	frequency	intensity
1	a'	193.0	5.7
2	a''	212.9	0.9
3	a'	500.1	2.4
4	a''	520.4	1.9
5	a'	601.8	49.2
6	a'	692.1	1.7
7	a''	705.4	28.7
8	a''	891.2	17.1
9	a'	945.8	2.3
10	a'	1107.9	6.5
11	a'	1269.8	41.2
12	a'	1708.0	5.0
13	a'	2163.8	10.6
14	a'	3261.4	1.1
15	a'	3457.0	71.1

Table S8: Harmonic vibrational frequencies (cm^{-1}) and IR intensities ($km\ mol^{-1}$) of isomer **3** of C_5H_2 calculated at the fc-CCSD(T)/cc-pVTZ level of theory.

Mode	symmetry	frequency	intensity
1	b_1	122.3	3.1
2	b_2	139.3	0.0
3	b_2	254.9	5.7
4	b_1	270.9	8.5
5	b_2	445.4	0.9
6	b_1	576.2	2.8
7	a_1	745.5	0.4
8	b_1	943.4	31.6
9	b_2	1030.3	0.4
10	a_1	1346.9	18.0
11	a_1	1503.5	7.7
12	a_1	1921.8	176.6
13	a_1	2151.6	677.1
14	a_1	3127.3	0.6
15	b_2	3215.1	0.0

Table S9: Harmonic vibrational frequencies (cm^{-1}) and IR intensities (km mol^{-1}) of isomer **4** of C_5H_2 calculated at the fc-CCSD(T)/cc-pVTZ level of theory.

Mode	symmetry	frequency	intensity
1	a_1	128.2	2.1
2	b_2	291.1	27.8
3	b_1	296.6	0.7
4	a_2	305.5	0.0
5	a_1	329.2	107.8
6	b_2	435.8	2.8
7	a_1	560.5	3.9
8	a_2	768.2	0.0
9	b_1	768.7	35.0
10	a_1	832.1	2.9
11	b_2	1274.3	88.2
12	b_2	1974.3	186.4
13	a_1	2025.1	1.2
14	b_2	3451.2	109.5
15	a_1	3453.8	25.8

Table S10: Harmonic vibrational frequencies (cm^{-1}) and IR intensities ($km\ mol^{-1}$) of isomer **5** of C_5H_2 calculated at the fc-CCSD(T)/cc-pVTZ level of theory.

Mode	symmetry	frequency	intensity
1	a'	128.8	3.3
2	a''	198.2	3.2
3	a'	286.6	2.1
4	a''	306.2	13.2
5	a'	569.2	4.2
6	a'	634.4	44.6
7	a''	675.6	32.4
8	a''	874.0	2.9
9	a'	947.8	50.8
10	a'	1149.5	4.8
11	a'	1383.3	4.0
12	a'	1989.5	582.7
13	a'	2137.5	82.6
14	a'	3133.9	0.6
15	a'	3454.1	58.5

Table S11: Harmonic vibrational frequencies (cm^{-1}) and IR intensities ($km\ mol^{-1}$) of isomer **6** of C_5H_2 calculated at the fc-CCSD(T)/cc-pVTZ level of theory.

Mode	symmetry	frequency	intensity
1	a'	66.8015	3.2536
2	a''	146.0506	1.3678
3	a''	320.7860	0.2191
4	a'	342.1422	35.3010
5	a'	391.7131	41.4646
6	a'	453.2695	31.5590
7	a''	582.3653	15.0567
8	a'	725.7820	53.8401
9	a''	731.6550	22.5675
10	a'	760.0319	150.5521
11	a'	1373.8075	2.5934
12	a'	1898.2314	33.0096
13	a'	2061.3028	7.3018
14	a'	3119.3874	16.5238
15	a'	3452.7960	109.8180

Table S12: Harmonic vibrational frequencies (cm^{-1}) and IR intensities (km mol^{-1}) of isomer **7** of C_5H_2 calculated at the fc-CCSD(T)/cc-pVTZ level of theory.

Mode	symmetry	frequency	intensity
1	b_u	472.5i	929.5
2	b_u	91.6i	4.2
3	a_u	144.8	1.7
4	b_g	320.6	0.0
5	b_u	376.2	6.7
6	a_g	441.3	0.0
7	a_g	530.6	0.0
8	a_u	590.0	8.0
9	a_g	771.2	0.0
10	a_u	816.5	28.9
11	b_u	1500.4	5.7
12	b_u	1785.7	23.4
13	a_g	1941.3	0.0
14	b_u	3329.4	43.9
15	a_g	3332.3	0.0

Table S13: Harmonic vibrational frequencies (cm^{-1}) and IR intensities ($km\ mol^{-1}$) of isomer **8** of C_5H_2 calculated at the fc-CCSD(T)/cc-pVTZ level of theory.

Mode	symmetry	frequency	intensity
1	b_1	147.8	1.4
2	b_2	161.9	0.3
3	b_1	469.7	10.7
4	b_2	485.1	0.4
5	a_1	726.6	1.8
6	b_1	765.0	45.5
7	a_2	933.1	0.0
8	b_2	939.9	11.1
9	a_1	978.4	1.9
10	b_2	1130.2	12.7
11	a_1	1445.7	163.7
12	a_1	1645.6	36.8
13	a_1	2051.5	1091.1
14	b_2	3253.2	8.6
15	a_1	3292.3	16.5

Table S14: Harmonic vibrational frequencies (cm^{-1}) and IR intensities (km mol^{-1}) of isomer **9** of C_5H_2 calculated at the fc-CCSD(T)/cc-pVTZ level of theory.

Mode	symmetry	frequency	intensity
1	b_1	251.7	2.3
2	b_2	330.4	0.5
3	b_1	589.7	3.7
4	b_2	617.3	44.9
5	a_2	707.6	0.0
6	a_1	782.7	0.0
7	b_1	877.1	45.7
8	b_2	952.6	6.0
9	a_1	1045.5	6.6
10	b_2	1064.6	0.8
11	a_1	1396.3	68.5
12	a_1	1453.9	1.4
13	a_1	1794.5	27.4
14	a_1	3180.5	1.9
15	b_2	3281.7	0.4

Table S15: Harmonic vibrational frequencies (cm^{-1}) and IR intensities ($km\ mol^{-1}$) of isomer **10** of C_5H_2 calculated at the fc-CCSD(T)/cc-pVTZ level of theory.

Mode	symmetry	frequency	intensity
1	b_2	82.7i	4.2
2	b_1	204.6	0.0
3	b_2	282.0	12.4
4	a_2	462.7	0.0
5	b_1	546.9	6.6
6	b_2	602.3	6.3
7	a_1	704.7	0.1
8	b_1	926.5	36.9
9	b_2	995.5	0.3
10	a_1	1244.5	2.7
11	a_1	1427.6	0.1
12	a_1	1531.3	4.4
13	a_1	1947.6	7.5
14	a_1	3123.5	0.0
15	b_2	3208.3	0.7

Table S16: Harmonic vibrational frequencies (cm^{-1}) and IR intensities ($km\ mol^{-1}$) of isomer **11** of C_5H_2 calculated at the fc-CCSD(T)/cc-pVTZ level of theory.

Mode	symmetry	frequency	intensity
1	b_u	164.1	49.9
2	a_u	232.9	23.2
3	b_u	419.4	40.4
4	a_u	450.4	3.4
5	a_g	542.2	0.0
6	b_g	749.6	0.0
7	a_u	819.6	59.2
8	a_g	839.7	0.0
9	b_u	959.2	10.2
10	a_g	1065.4	0.0
11	b_u	1139.2	26.7
12	a_g	1576.2	0.0
13	b_u	1680.3	4.9
14	a_g	3288.2	0.0
15	b_u	3290.6	24.5

Table S17: Harmonic vibrational frequencies (cm^{-1}) and IR intensities ($km\ mol^{-1}$) of isomer **12** of C_5H_2 calculated at the fc-CCSD(T)/cc-pVTZ level of theory.

Mode	symmetry	frequency	intensity
1	e'	490.7	49.9
2	e'	490.7	49.9
3	e''	777.1	0.0
4	e''	777.1	0.0
5	e'	839.4	20.4
6	e'	839.4	20.4
7	$a1'$	846.8	0.0
8	e''	1011.5	0.0
9	e''	1011.5	0.0
10	e'	1129.0	8.3
11	e'	1129.0	8.3
12	$a2''$	1276.2	19.6
13	$a1'$	1299.7	0.0
14	$a2''$	3330.0	12.3
15	$a1'$	3331.6	0.0

Table S18: Harmonic vibrational frequencies (cm^{-1}) and IR intensities (km mol^{-1}) of isomer **13** of C_5H_2 calculated at the fc-CCSD(T)/cc-pVTZ level of theory.

Mode	symmetry	frequency	intensity
1	a_2	207.5	0.0
2	a_1	244.1	2.3
3	b_2	321.0	23.6
4	b_1	444.3	4.2
5	a_1	593.3	13.2
6	a_2	758.3	0.0
7	b_1	791.5	56.4
8	b_2	944.3	4.2
9	a_1	975.1	37.5
10	b_2	1013.7	11.5
11	a_1	1064.7	4.7
12	a_1	1571.2	31.2
13	b_2	1643.5	1.6
14	a_1	3280.2	14.9
15	b_2	3284.6	0.7

Table S19: Harmonic vibrational frequencies (cm^{-1}) and IR intensities ($km\ mol^{-1}$) of isomer **14** of C_5H_2 calculated at the fc-CCSD(T)/cc-pVTZ level of theory.

Mode	symmetry	frequency	intensity
1	a''	104.1	0.0
2	a'	110.6	7.9
3	a'	341.5	11.5
4	a''	402.7	1.9
5	a''	709.8	21.0
6	a'	748.6	0.1
7	a''	891.0	17.2
8	a'	934.6	4.1
9	a'	997.6	9.3
10	a'	1172.5	35.2
11	a'	1288.7	34.4
12	a'	1637.3	52.3
13	a'	1756.9	104.9
14	a'	3127.7	20.0
15	a'	3267.1	1.6

Table S20: Harmonic vibrational frequencies (cm^{-1}) and IR intensities ($km\ mol^{-1}$) of isomer **15** of C_5H_2 calculated at the fc-CCSD(T)/cc-pVTZ level of theory.

Mode	symmetry	frequency	intensity
1	b_2	236.3i	0.1
2	a_2	115.9	0.0
3	b_1	427.1	4.3
4	b_2	468.2	0.0
5	a_1	664.5	9.2
6	b_1	757.2	45.5
7	b_2	879.7	0.0
8	a_2	898.0	0.0
9	a_1	971.8	2.5
10	b_2	1043.8	26.2
11	a_1	1172.2	107.4
12	a_1	1624.6	1.8
13	a_1	1656.9	34.4
14	b_2	3252.6	9.3
15	a_1	3300.2	7.3

Table S21: Rotational and centrifugal distortion constants (in MHz), and inertial axis dipole moment components (in Debye; $\perp^r$ representation) of isomers **13** and **14** of C_5H_2 calculated at the fc-CCSD(T)/cc-pVTZ level of theory.

Isomer[a]	A_e	B_e	C_e	Δ_J	Δ_K	Δ_{JK}	δ_J	δ_K	μ_a	μ_b	$\lvert\mu\rvert$
**13**	18897.05	5445.32	4227.22	0.1185×10^{-2}	-0.9819×10^{-2}	0.2280×10^{-1}	0.2785×10^{-3}	0.1402×10^{-1}	5.1731	-	5.1731
**14**	22907.74	4091.85	3471.72	0.5571×10^{-2}	0.5168	-0.8922×10^{-1}	-0.1446×10^{-2}	-0.6344×10^{-4}	0.0164	-4.4903	4.4903

[a] For isomers **1-9**, please see *J. Phys. Chem. A* **2019**, 123, 6618-6627. It is noted here that isomers **10** and **15** are transition states whereas isomer **7** is a second-order saddle point. Therefore, spectroscopic parameters for these systems are not given. For isomers **11** and **12**, there is no permanent dipole. Thus, they are not suitable molecules for rotational spectroscopy. [b] Centrifugal distortion constants are from the S-reduced Hamiltonian for **14** considering the fact that it is approaching close to the prolate limit.

Table S22: Theoretical Equilibrium Rotational Constants (A_e, B_e, C_e; in MHz) Obtained at the CCSD(T) Level Using Different Basis Sets (Obtained With and Without the Frozen-Core Approximation) Along With Experimentally Measured Rotational Constants (A_0, B_0, C_0; in MHz) for Isomers **2**, **3**, **5** of C_5H_2 (see next Table for isomer **8**)

		Rotational constants			% Error		
Isomer	Level of theory	A_e	B_e	C_e			
**2**	Semi-Experimental[e]	36972.92	3448.64	3154.31	-	-	-
	PBE-D3BJ	34776.97	3410.30	3105.75	6.31	1.12	1.56
	BLYP-D3BJ	34772.95	3406.65	3102.68	6.33	1.23	1.66
	M06-L	35357.19	3460.34	3151.87	4.57	-0.34	0.08
	TPSS-D3BJ	34832.08	3421.18	3115.20	6.15	0.80	1.26
	B3LYP-D3BJ	35276.94	3450.69	3143.23	4.81	-0.06	0.35
	PBE0-D3	35266.57	3452.84	3144.93	4.84	-0.12	0.30
	BMK-D3BJ	34305.88	3417.45	3107.86	7.77	0.91	1.49
	M06-2X	35256.00	3448.04	3140.87	4.87	0.02	0.43
	ωB97X-D	35291.05	3455.20	3147.08	4.77	-0.19	0.23
	CAM-B3LYP-D3BJ	35545.72	3467.14	3159.01	4.02	-0.53	-0.15
	B2-PLYP-D3BJ	35080.47	3437.28	3130.54	5.39	0.33	0.76
	DSD-PBEP86-D3BJ	34906.49	3425.77	3119.61	5.92	0.67	1.11
**3**	Semi-Experimental[e]	231551.05	2216.88	2283.64	-	-	-
	PBE-D3BJ	288084.24	2289.71	2271.66	N/A	-3.18	0.53
	BLYP-D3BJ	290215.02	2292.37	2274.41	N/A	-3.29	0.41
	M06-L	294818.00	2323.38	2305.21	N/A	-4.58	-0.94
	TPSS-D3BJ	291046.32	2299.95	2281.92	N/A	-3.61	0.08
	B3LYP-D3BJ	293043.81	2325.07	2306.77	N/A	-4.65	-1.00
	PBE0-D3	290928.94	2323.77	2305.36	N/A	-4.60	-0.94
	BMK-D3BJ	290741.94	2324.26	2305.82	N/A	-4.62	-0.96
	M06-2X	291808.85	2325.92	2307.52	N/A	-4.69	-1.03
	ωB97X-D	291451.07	2332.41	2313.89	N/A	-4.95	-1.31
	CAM-B3LYP-D3BJ	292506.42	2342.33	2323.72	N/A	-5.36	-1.72
	B2-PLYP-D3BJ	293012.38	2313.86	2295.74	N/A	-4.19	-0.53
	DSD-PBEP86-D3BJ	291090.37	2305.76	2287.64	N/A	-3.85	-0.17
**5**	Semi-Experimental[e]	31151.23	2863.13	2621.54	-	-	-
	PBE-D3BJ	31828.92	2844.85	2611.44	-2.13	0.64	0.39
	BLYP-D3BJ	31858.95	2844.08	2610.99	-2.22	0.67	0.40
	M06-L	32808.78	2871.76	2640.62	-5.05	-0.30	-0.72
	TPSS-D3BJ	31747.91	2858.87	2622.70	-1.88	0.15	-0.04
	B3LYP-D3BJ	32319.76	2881.41	2645.55	-3.62	-0.63	-0.91
	PBE0-D3	32255.27	2883.33	2646.73	-3.42	-0.70	-0.95
	BMK-D3BJ	31631.84	2889.51	2647.65	-1.52	-0.91	-0.99
	M06-2X	31802.01	2890.22	2649.44	-2.05	-0.94	-1.05
	ωB97X-D	31949.95	2893.95	2653.59	-2.50	-1.06	-1.21
	CAM-B3LYP-D3BJ	32265.81	2902.36	2662.84	-3.45	-1.35	-1.55
	B2-PLYP-D3BJ	31903.20	2873.29	2635.89	-2.36	-0.35	-0.54
	DSD-PBEP86-D3BJ	31573.48	2867.58	2628.82	-1.34	-0.16	-0.28
	Measured[d]	31979(6)	3530.13	3176.84	-	-	-

Table S23: Theoretical Equilibrium Rotational Constants (A_e, B_e, C_e; in MHz) Obtained at the CCSD(T) Level Using Different Basis Sets (Obtained With and Without the Frozen-Core Approximation) Along With Experimentally Measured Rotational Constants (A_0, B_0, C_0; in MHz) for Isomer **8** of C_5H_2

		Rotational constants			% Error		
Isomer	Level of theory	A_e	B_e	C_e			
**8**	Semi-Experimental[e]	34494.31	3515.75	3202.60	-	-	-
	PBE-D3BJ	31895.10	3495.39	3150.17	8.15	0.58	1.66
	BLYP-D3BJ	31951.11	3492.37	3148.26	7.96	0.67	1.73
	M06-L	32469.73	3550.52	3200.55	6.24	-0.98	0.06
	TPSS-D3BJ	32043.94	3507.76	3161.66	7.65	0.23	1.29
	B3LYP-D3BJ	32383.88	3549.26	3198.69	6.52	-0.94	0.12
	PBE0-D3	32335.21	3554.21	3202.23	6.68	-1.08	0.01
	BMK-D3BJ	31945.51	3514.90	3166.50	7.98	0.02	1.14
	M06-2X	32409.92	3557.06	3205.27	6.43	-1.16	-0.08
	ωB97X-D	32460.20	3561.98	3209.76	6.27	-1.30	-0.22
	CAM-B3LYP-D3BJ	32576.31	3580.04	3225.56	5.89	-1.80	-0.71
	B2-PLYP-D3BJ	32223.32	3538.94	3188.73	7.05	-0.66	0.43
	DSD-PBEP86-D3BJ	32086.32	3531.32	3181.20	7.50	-0.44	0.67

[a] Experimental A_0, B_0, and C_0 values are converted to semi-experimental A_e, B_e, and C_e values using vibrational corrections calculated at the B3LYP/jun-cc-pVTZ level of theory. [b] Experimental A_0, B_0, and C_0 values are converted to semi-experimental A_e, B_e, and C_e values using vibrational corrections calculated at the B3LYP/jun-cc-pVTZ level of theory. [c] This is not a measured value. This value has been derived assuming a planar structure, that is, 1/C - 1/A - 1/B = 0. [d] Experimental A_0, B_0, and C_0 values are converted to semi-experimental A_e, B_e, and C_e values using vibrational corrections calculated at the B3LYP/jun-cc-pVTZ level of theory.
